# Computational Analysis of the Global Effects of *Ly6E* in the Immune Response to Coronavirus Infection Using Gene Networks

**DOI:** 10.3390/genes11070831

**Published:** 2020-07-21

**Authors:** Fernando M. Delgado-Chaves, Francisco Gómez-Vela, Federico Divina, Miguel García-Torres, Domingo S. Rodriguez-Baena

**Affiliations:** Pablo de Olavide University, Carretera de Utrera km 1, ES-41013 Seville, Spain; fgomez@upo.es (F.G.-V.); fdiv@upo.es (F.D.); mgarciat@upo.es (M.G.-T.); dsrodbae@upo.es (D.S.R.-B.)

**Keywords:** gene co-expression network, murine coronavirus, viral infection, immune response, data mining, systems biology

## Abstract

Gene networks have arisen as a promising tool in the comprehensive modeling and analysis of complex diseases. Particularly in viral infections, the understanding of the host-pathogen mechanisms, and the immune response to these, is considered a major goal for the rational design of appropriate therapies. For this reason, the use of gene networks may well encourage therapy-associated research in the context of the coronavirus pandemic, orchestrating experimental scrutiny and reducing costs. In this work, gene co-expression networks were reconstructed from RNA-Seq expression data with the aim of analyzing the time-resolved effects of gene *Ly6E* in the immune response against the coronavirus responsible for murine hepatitis (MHV). Through the integration of differential expression analyses and reconstructed networks exploration, significant differences in the immune response to virus were observed in *Ly6E*${}^{\Delta HSC}$ compared to *wild type* animals. Results show that *Ly6E* ablation at hematopoietic stem cells (HSCs) leads to a progressive impaired immune response in both liver and spleen. Specifically, depletion of the normal leukocyte mediated immunity and chemokine signaling is observed in the liver of *Ly6E*${}^{\Delta HSC}$ mice. On the other hand, the immune response in the spleen, which seemed to be mediated by an intense chromatin activity in the normal situation, is replaced by ECM remodeling in *Ly6E*${}^{\Delta HSC}$ mice. These findings, which require further experimental characterization, could be extrapolated to other coronaviruses and motivate the efforts towards novel antiviral approaches.

## 1. Introduction

The recent SARS-CoV-2 pandemic has exerted an unprecedented pressure on the scientific community in the quest for novel antiviral approaches. A major concern regarding SARS-CoV-2 is the capability of the *coronaviridae* family to cross the species barrier and infect humans [1]. This, along with the tendency of coronaviruses to mutate and recombine, represents a significant threat to global health, which ultimately has put interdisciplinary research on the warpath towards the development of a vaccine or antiviral treatments.

Given the similarities found amongst the members of the *coronaviridae* family [2,3], analyzing the global immune response to coronaviruses may shed some light on the natural control of viral infection, and inspire prospective treatments. This may well be achieved from the perspective of systems biology, in which the interactions between the biological entities involved in a certain process are represented by means of a mathematical system [4]. Within this framework, gene networks (GN) have become an important tool in the modeling and analysis of biological processes from gene expression data [5]. GNs constitute an abstraction of a given biological reality by means of a graph composed by nodes and edges. In such a graph, nodes represent the biological elements involved (i.e., genes, proteins or RNAs) and edges represent the relationships between the nodes. In addition, GNs are also useful to identify genes of interest in biological processes, as well as to discover relationships among these. Thus, they provide a comprehensive picture of the studied processes [6,7].

Among the different types of GNs, gene co-expression networks (GCNs) are widely used in the literature due to their computational simplicity and good performance in order to study biological processes or diseases [8,9,10]. GCNs usually compute pairwise co-expression indices for all genes. Then, the level of interaction between two genes is considered significant if its score is higher than a certain threshold, which is set *ad hoc*. Traditionally, statistical-based co-expression indices have been used to calculate the dependencies between genes [5,7]. Some of the most popular correlation coefficients are Pearson, Kendall or Spearman [11,12,13]. Despite their popularity, statistical-based measures present some limitations [14]. For instance, they are not capable of identifying non-linear interactions and the dependence on the data distribution in the case of parametric correlation coefficients. In order to overcome some of these limitations, new approaches, e.g., the use of information theory-based measures or ensemble approaches, are receiving much attention [15,16,17].

Gene Co-expression Networks (GCNs) have already been applied to the study of dramatic impact diseases, such as cancer [18], diabetes [19] or viral infections (e.g., HIV) in order to study the role of immune response to these illnesses [20,21]. Genetic approaches are expected to be the best strategy to understand viral infection and the immune response to it, potentially identifying the mechanisms of infection and assisting the design of strategies to combat infection [22,23]. The current gene expression profiling platforms, in combination with high-throughput sequencing, can provide time-resolved transcriptomic data, which can be related to the infection process. The main objective of this approach is to generate knowledge on the immune functioning upon viral entry into the organism, which means mean a perturbation to the system.

In the context of viral infection, a first defense line is the innate response mediated by interferons, a type of cytokines which eventually leads to the activation of several genes of antiviral function [24]. Globally, these genes are termed interferon-stimulated genes (ISGs), and regulate processes like inflammation, chemotaxis or macrophage activation among others. Furthermore, ISGs are also involved in the subsequent acquired immune response, specific for the viral pathogen detected [25]. Gene *Ly6E* (lymphocyte antigen 6 family member e), which has been related to T cell maturation and tumorogenesis, is amongst the ISGs [26]. This gene is transcriptionally active in a variety of tissues, including liver, spleen, lung, brain, uterus and ovary. Its role in viral infection has been elusive due to contradictory findings [27]. For example, in Liu et al. [28], *Ly6E* was associated with the resistance to Marek’s disease virus (MDV) in chickens. Moreover, differences in the immune response to mouse adenovirus type 1 (MAV-1) have been attributed to *Ly6E* variants [29]. Conversely, *Ly6E* has also been related to an enhancement of human immunodeficiency viruses (HIV-1) pathogenesis, by promoting HIV-1 entry through virus–cell fusion processes [30]. Also in the work by Mar et al. [31], the loss of function of *Ly6E* due to gene *knockout* reduced the infectivity of Influenza A virus (IAV) and yellow fever virus (YFV). This enhancing effect of *Ly6E* on viral infection has also been observed in other enveloped RNA viruses such as in West Nile virus (WNV), dengue virus (DEN), Zika virus (ZIKV), O’nyong nyong virus (ONNV) and Chikungunya virus (CHIKV) among others [32]. Nevertheless, the exact mechanisms through which *Ly6E* modulates viral infection virus-wise, and sometimes even cell type-dependently, require further characterization.

In this work we present a time-resolved study of the immune response of mice to a coronavirus, the murine hepatitis virus (MHV), in order to analyze the implications of gene *Ly6E*. To do so, we have applied a GCN reconstruction method called *EnGNet* [33], which is able to perform an ensemble strategy to combine three different co-expression measures, and a topology optimization of the final network. *EnGNet* has outscored other methods in terms of network precision and reduced network size, and has been proven useful in the modeling of disease, as in the case of Human post-traumatic stress disorder.

The rest of the paper is organized as follows. In the next section, we propose a description of related works. In Section 3, we first describe the dataset used in this paper, and then we introduce the *EnGNet* algorithm and the different methods used to infer and analyze the generated networks. The results obtained are detailed in Section 4, while, in Section 5, we propose a discussion of the results presented in the previous section. Finally, in Section 6, we draw the main conclusions of our work.

## 2. Related Works

As already mentioned, gene co-expression networks have been extensively applied in the literature for the understanding of the mechanisms underlying complex diseases like cancer, diabetes or Alzheimer [34,35,36]. Globally, GCN serve as an *in silico* genetic model of these pathologies, highlighting the main genes involved in these at the same time [37]. Besides, the identification of modules in the inferred GCNs, may lead to the discovery of novel biomarkers for the disease under study, following the ’guilt by association’ principle. Along these lines, GCNs are also considered suitable for the study of infectious diseases, as those caused by viruses to the matter at hand [38]. To do so, multiple studies have analyzed the effects of viral infection over the organism, focusing on immune response or tissue damage [39,40].

For instance, the analysis of gene expression using co-expression networks is shown in the work by Pedragosa et al. [41], where the infection caused by Lymphocytic Choriomeningitis Virus (LCMV) is studied over time in mice spleen using GCNs. In Ray et al. [42], GCNs are reconstructed from different microarray expression data in order to study HIV-1 progression, revealing important changes across the different infection stages. Similarly, in the work presented by McDermott et al. [43], the over- and under-stimulation of the innate immune response to severe acute respiratory syndrome coronavirus (SARS-CoV) infection is studied. Using several network-based approaches on multiple *knockout* mouse strains, authors found that ranking genes based on their network topology made accurate predictions of the pathogenic state, thus solving a classification problem. In [39], co-expression networks were generated by microarray analysis of pediatric influenza-infected samples. Thanks to this study, genes involved in the innate immune system and defense to virus were revealed. Finally, in the work by Pan et al. [44], a co-expression network is constructed based on differentially-expressed microRNAs and genes identified in liver tissues from patients with hepatitis B virus (HBV). This study provides new insights on how microRNAs take part in the molecular mechanism underlying HBV-associated acute liver failure.

The alarm posed by the COVID-19 pandemic has fueled the development of effective prevention and treatment protocols for 2019-nCoV/SARS-CoV-2 outbreak [45]. Due to the novelty of SARS-CoV-2, recent research takes similar viruses, such as SARS-CoV and Middle East Respiratory Syndrome coronavirus (MERS-CoV), as a starting point. Other coronaviruses, like Mouse Hepatitis Virus (MHV), are also considered appropriate for comparative studies in animal models, as demonstrated in the work by De Albuquerque et al. [46] and Ding et al. [47]. MHV is a murine coronavirus (M-CoV) that causes an epidemic illness with high mortality, and has been widely used for experimentation purposes. Works like the ones by Case et al. [48] and Gorman et al. [49], study the innate immune response against MHV arbitrated by interferons, and those interferon-stimulated genes with potential antiviral function. This is the case of gene *Ly6E*, which has been shown to play an important role in viral infection, as well as various orthologs of the same gene [50,51]. Mechanistic approaches often involved the ablation of the gene under study, like in the work by Mar et al. [31], where gene *knockout* was used to characterize the implications of *Ly6E* in Influenza A infection. As it is the case of Giotis et al. [52], these studies often involve global transcriptome analyses, via RNA-seq or microarrays, together with computational efforts, which intend to screen the key elements of the immune system that are required for the appropriate response. This approach ultimately leads experimental research through predictive analyses, as in the case of co-expression gene networks [53].

## 3. Materials and Methods

In the following subsections, the main methods and GCN reconstruction steps are addressed. First, in Section 3.1, the original dataset used in the present work is described, together with the experimental design. Then, in Section 4.1, the data preprocessing steps are described. Subsequently in Section 3.3, key genes controlling the infection progression are extracted through differential expression analyses. Finally, the inference of GCNs and their analysis are detailed in Section 3.4 and Section 3.5, respectively.

### 3.1. Original Dataset Description

The original experimental design can be described as follows. The progression of the MHV infection at genetic level was evaluated in two genetic backgrounds: wild type (*wt*, Ly6Efl/fl) and Ly6E *knockout* mutants (*ko*, *Ly6E^*Δ*HSC^*). The ablation of gene *Ly6E* in all cell types is lethal, hence the *Ly6E^*Δ*HSC^* strain contains a disrupted version of gene Ly6E only in hematopoietic stem cells (HSC), which give rise to myeloid and lymphoid progenitors of all blood cells. *Wild type* and *Ly6E^*Δ*HSC^* mice were injected intraperitoneally with 5000 PFU MHV-A59. At 3 and 5 days post-injection (d p.i.), mice were euthanized and biological samples for RNA-Seq were extracted. The overall effects of MHV infection in both *wt* and *ko* strains was assessed in liver and spleen.

In total 36 samples were analyzed, half of these corresponding to liver and spleen, respectively. From the 18 organ-specific samples, 6 samples correspond to mock infection (negative control), 6 to MHV-infected samples at 3 d p.i. and 6 to MHV-infected samples at 5 d p.i. For each sample, two technical replicates were obtained. Libraries of cDNA generated from the samples were sequenced using Illumina NovaSeq 6000. Further details on sample preparation can be found in the original article by Pfaender et al. [54]. For the sake of simplicity, MHV-infected samples at 3 and 5 d p.i. will be termed ’cases’, whereas mock-infection samples will be termed ’controls’.

The original dataset consists of 72 files, one per sample replicate, obtained upon the mapping of the transcript reads to the reference genome. Reads were recorded in three different ways, considering whether these mapped introns, exons or total genes. Then, a count table was retrieved from these files by selecting only the total gene counts of each sample replicate file.

### 3.2. Data Pre-Processing

Pre-processing was performed using the EdgeR [55] R package. The original dataset by Pfaender et al. [54] was retrieved from GEO (accession ID: GSE146074) using the *GEOquery* [56] package. Additional files on sample information and treatment were also used to assist the modeling process.

By convention, a sequencing depth per gene below 10 is considered neglectable [57,58]. Genes meeting this criterion are known as low expression genes, and are often removed since they add noise and computational burden to the following analyses [59]. In order to remove genes showing less than 10 reads across all conditions, counts per million (CPM) normalization was performed, so possible differences between library sizes for both replicates would not affect the result.

Afterwards, Principal Components Analyses (PCA) were performed over the data in order to detect the main sources of variability across samples. PCA were accompanied by unsupervised k-medoid clustering analyses, in order to identify different groups of samples. In addition, multidimensional scaling plots (MDS) were applied to further separate samples according to their features. Last, between-sample similarities were assessed through hierarchical clustering.

### 3.3. Differential Expression Analyses

The analyses of differential expression served a two-way purpose, (i) the exploration of the directionality in the gene expression changes upon viral infection, and (ii) the identification of key regulatory elements for the subsequent network reconstruction. In the present application, differentially-expressed genes (DEG) were filtered from the original dataset and proceeded to the reconstruction process. This approximation enabled the modeling of the genetic relationships that are considered of relevance in the presented comparison [60,61,62]. In the present work mice samples were compared organ-wise depending on whether these corresponded to control, 3 d p.i. and 5 d p.i.

The identification of DEG was performed using the Limma [63] R package, which provides non-parametric robust estimation of the gene expression variance. This package includes Voom, a method that incorporates RNA-Seq count data into the Limma workbench, originally designed for microarrays [64]. In this case, a minimum log2-fold-change (log2FC) of 2 was chosen, which corresponds to four fold changes in the gene expression level. P-value was adjusted by Benjamini-Hochberg [65] and the selected adjusted p-value cutoff was 0.05.

### 3.4. Inference of the Gene Networks: *EnGNet*

In order to generate gene networks the *EnGNet* algorithm was used. This technique, presented in Gómez-Vela et al. [33], is able to compute gene co-expression networks with a competitive performance compared other approaches from the literature. *EnGNet* performs a two-step process to infer gene networks: (a) an ensemble strategy for a reliable co-expression networks generation, and (b) a greedy algorithm that optimizes both the size and the topological features of the network. These two features of *EnGNet* offer a reliable solution for generating gene networks. In fact, *EnGNet* relies on three statistical measures in order to obtain networks. In particular, the measures used are the Spearman, Kendall and normalized mutual information (NMI), which are widely used in the literature for inferring gene networks. *EnGNet* uses these measures simultaneously by applying an ensemble strategy based on major voting, i.e., a relationship will be considered correct if at least 2 of the 3 measures evaluate the relationship as correct. The evaluation is based on different independent thresholds. In this work, the different thresholds were set to the values originally used in [33]: 0.9, 0.8 and 0.7 for Spearman, Kendall and NMI, respectively.

In addition, as mentioned above, *EnGNet* performs an optimization of the topological structure of the networks obtained. This reduction is based on two steps: (i) the pruning of the relations considered of least interest in the initial network, and (ii) the analysis of the hubs present in the network. For this second step of the final network reconstruction, we have selected the same threshold that was used in [33], i.e., 0.7. Through this optimization, the final network produced by *EnGNet* results easier to analyze computationally, due to its reduced size.

### 3.5. Networks Analyses

Networks were imported to R for the estimation of topology parameters and the addition of network features that are of interest for the latter network analysis and interpretation. These attributes were added to the reconstructed networks to enrich the modeling using the *igraph* [66] R package. The networks were then imported into *Cytoscape* [67] through RCy3 [68] for examination and analyses purposes. In this case, two kind of analyses were performed: (i) a topological analysis and (ii) an enrichment analysis.

Regarding the topological analysis, clustering evaluation was performed in order to identify densely connected nodes, which, according to the literature, are often involved in a same biological process [69]. The chosen clustering method was community clustering (GLay) [70], implemented via *Cytoscape*’s *ClusterMaker* app [71], which has yielded significant results in the identification of densely connected modules [72,73]. Among the topology parameters, *degree* and *edge betweenness* were estimated. The *degree* of a node refers to the number of its linking nodes. On the other hand, the *betweenness* of an edge refers to the number of shortest paths which go through that edge. Both parameters are considered as a measure of the implications of respectively nodes and edges in a certain network. Particularly, nodes whose *degree* exceeds the average network node *degree*, the so called *hubs*, are considered key elements of the biological processes modeled by the network. In this particular case, the distribution of nodes’ degree network was analyzed so those nodes whose degree exceeded a threshold were selected as hubs. This threshold is defined as Q3+1.5×IQR, where *Q*3 is the third quartile and *IQR* the interquartile range of the degree distribution. This method has been widely used for the detection of upper outliers in non-parametric distributions [74,75], as it is the case. However, the outlier definition does not apply to this distribution since those nodes whose degree are far above the median degree are considered hubs.

On the other hand, Gene Ontology (GO) Enrichment Analysis provides valuable insights on the biological reality modeled by the reconstructed networks. The Gene Ontology Consortium [76] is a data base that seeks for a unified nomenclature for biological entities. GO has developed three different ontologies, which describe gene products in terms of the biological processes, cell components or molecular functions in which these are involved. Ontologies are built out of GO terms or annotations, which provide biological information of gene products. In this case, the *ClusterProfiler* [77] R package, allowed the identification of the statistically over-represented GO terms in the gene sets of interest. Additional enrichment analyses were performed using *DAVID* [78]. For both analyses, the complete genome of *Mus musculus* was selected as background. Finally, further details on the interplay of the genes under study was examined using the *STRING* database [79].

## 4. Results

**Figure 1:**
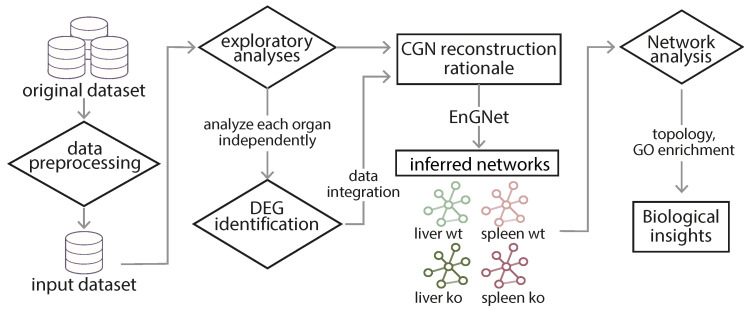
General scheme for the reconstruction method. The preprocessed data was subjected to exploratory and differential expression analyses, which imposed the reconstruction rationale. Four groups of samples were used to generate four independent networks, respectively modeling the immune response in the liver, both in the *wt* and the *ko* situations; and in the spleen, also in the *wt* and the *ko* scenarios.

The reconstruction of gene networks that adequately model viral infection involves multiple steps, which ultimately shape the final outcome. First, in Section 4.1, exploratory analyses and data preprocessing are detailed, which prompted the modeling rationale. Then, in Section 4.2, differential expression is evaluated for the samples of interest. Finally, networks reconstruction and analysis are addressed in Section 4.3. At the end, four networks were generated, both in an organ- and genotype-wise manner. A schematic representation of the GCN reconstruction approach is shown in Figure 1.

### 4.1. Data Pre-Processing and Exploratory Analyses

In order to remove low expression genes, a sequencing depth of 10 was found to correspond to an average CPM of 0.5, which was selected as threshold. Hence, genes whose expression was found over 0.5 CPM in at least two samples of the dataset were maintained, ensuring that only genes which are truly being expressed in the tissue will be studied. The dataset was Log2-normalized with priority to the following analyses, in accordance to the recommendations posed in Law et al. [64].

**Figure 2:**
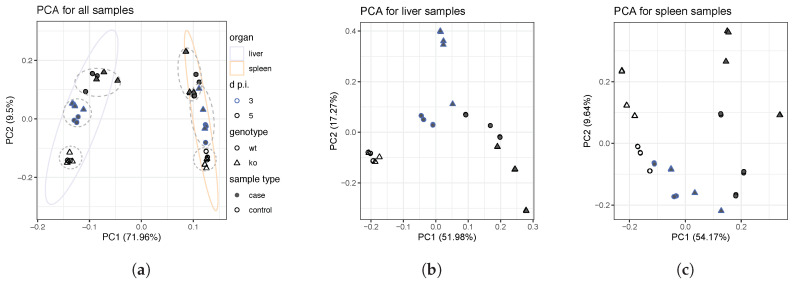
(**a**) PCA plot of the Log2-normalized counts for the exploratory analysis of all samples under study. The metric used for k-medoid partitioning was the Euclidean distance. Both replicates are included. Two groups, respectively corresponding to liver and spleen samples, are clearly differentiated. Dashed lines were added for improved visualization of the different groups that are distinguished within each organ. Organ-specific PCA for (**b**) liver and (**c**) spleen samples. Both replicates are included. PCA suggests the progressive nature of the MHV infection, where groups corresponding to mock infections, 3 d p.i. and 5 d p.i. are distinguished in varying degrees. Differences between controls and cases are more evident in liver samples. Figure 2a legend is the same for Figure 2b,c.

**Figure A1:**
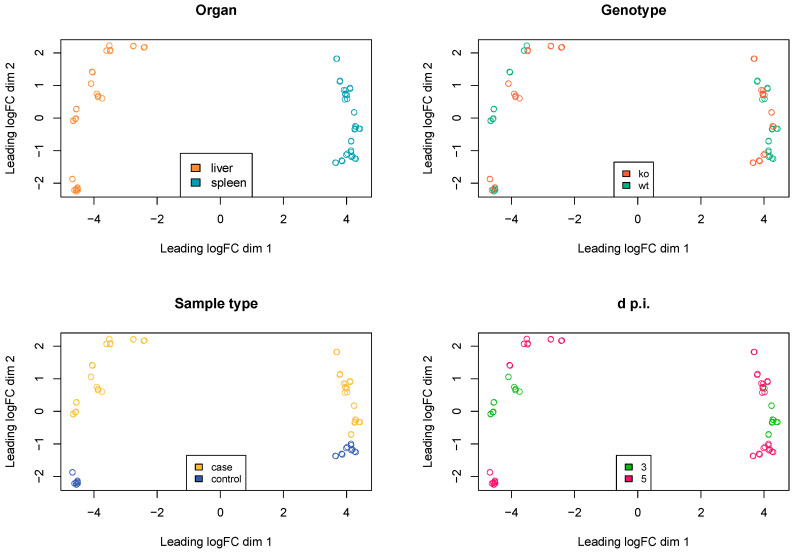
Multidimensional Scaling (MDS) plots showing main differences between individual samples according to the four features these present: organ procedence, genotype, sample type (mock infection or MHV-infected) and days post injection.

The results of both PCA and k-medoid clustering are shown in Figure 2a. Clustering of the Log2-normalized samples revealed clear differences between liver and spleen samples. Also, for each organ, three subgroups of analogous samples that cluster together are identified. These groups correspond to mock infection, MHV-infected mice at 3 d p.i. and MHV-infected mice at 5 d p.i. (dashed lines in Figure 2a). Finally, subtle differences were observed in homologous samples of different genotypes (Figure A1).

**Figure A2:**
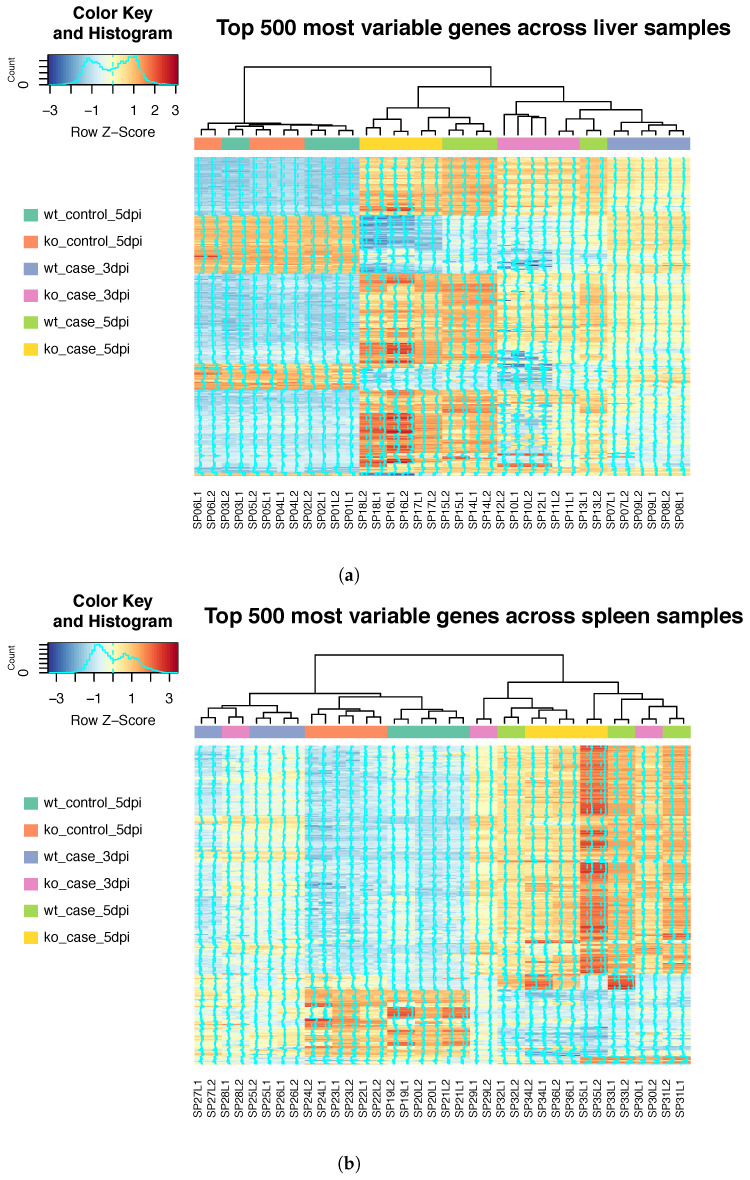
Top 500 most variable genes in (**a**) liver and (**b**) spleen samples. Log2-normalization was applied over the Counts per Million (CPMs) in order to properly compare distributions. Variance estimation reaffirms the homogenity of control vs. case samples. Overall, differences are also observed between 3 and 5 d p.i. case samples.

Organ-specific PCA revealed major differences between MHV-infected samples for *Ly6E*${}^{\Delta HSC}$ and *wt* genotypes, at both 3 and 5 d p.i. These differences were not observed in the mock infection (control situation). Organ-wise PCA are shown in Figure 2b,c. The distances between same-genotype samples illustrate the infection-prompted genetic perturbation from the uninfected status (control) to 5 d p.i., where clear signs of hepatitis were observed according to the original physiopathology studies [54]. On the other hand, the differences observed between both genotypes are indicative of the role of gene *Ly6E* in the appropriate response to viral infection. These differences are subtle in control samples, but in case samples, some composition biass is observed depending on whether these are *ko* or *wt*, especially in spleen samples. The comparative analysis of the top 500 most variable genes confirmed the differences observed in the PCA, as shown in Figure A2. Among the four different features of the samples under study: organ, genotype, sample type (case or control) and days post injection; the dissimilarities in terms of genotype were the subtlest.

In the light of these exploratory findings, the network reconstruction approach was performed as follows. Networks were reconstructed organ-wise, as these exhibit notable differences in gene expression. Additionally, a main objective of the present work is to evaluate the differences in the genetic response in the *wt* situation compared to the *Ly6E*${}^{\Delta HSC}$ *ko* background, upon the viral infection onset in the two mentioned tissues.

For each organ, Log2-normalized samples were coerced to generate time-series-like data, i.e., for each genotype, 9 samples will be considered as a set, namely 3 control samples, 3 case samples at 3 d p.i. and 3 case samples at 5 d p.i. Both technical replicates were included. This rational design seeks for a gene expression span representative of the infection progress. Thereby, control samples may well be considered as a time zero for the viral infection, followed by the corresponding samples at 3 and 5 d p.i. The proposed rationale is supported by the exploratory findings, which position 3 d p.i. samples between control and 5 d p.i. samples. At the same time, the reconstruction of gene expression becomes robuster with increasing number of samples. In this particular case, 18 measuring points are attained for the reconstruction of each one of the four intended networks, since two technical replicates were obtained per sample [80].

### 4.2. Identification of Differentially-Expressed Genes Between Wild Type and Ly6E${}^{\Delta HSC}$ Samples

The differential expression analyses were performed over the four groups of 9 samples explained above, with the aim of examining the differences in the immune response between *Ly6E*${}^{\Delta HSC}$ and *wt* samples. Limma - Voom differential expression analyses were performed over the Log2-normalized counts, in order to evaluate the different genotypes whilst contrasting the three infection stages: control vs. cases at 3 d p.i., control vs. cases at 5 d p.i. and cases at 3 vs. 5 d p.i. The choice of a minimum absolute log2FC ≥ 2, enabled considering only those genes that truly effect changes between *wt* and *Ly6E*${}^{\Delta HSC}$ samples, whilst maintaining a relatively computer-manageable number of DEG for network reconstruction. The latter is essential for the yield of accurate network sparseness values, as this is a main feature of gene networks [5].

**Figure 3:**
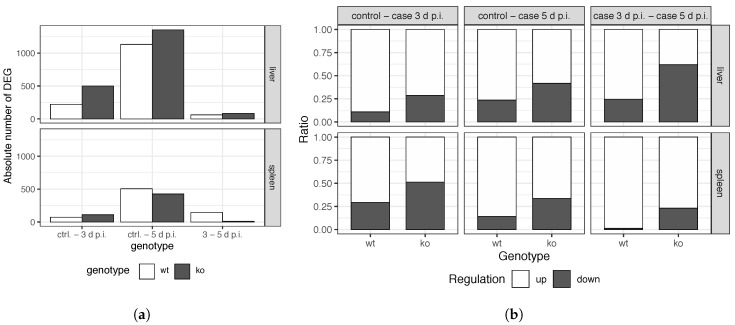
(**a**) Absolute numbers of DEG in the different comparisons (**b**) Ratio of up- and downregulated DEG in the different performed comparisons. Three comparisons were performed: control vs. case samples at 3 d p.i., control vs. case samples at 5 d p.i. and case samples at 3 vs. 5 d p.i. *ko* refers to *Ly6E^ΔHSC^* samples.

**Figure 4:**
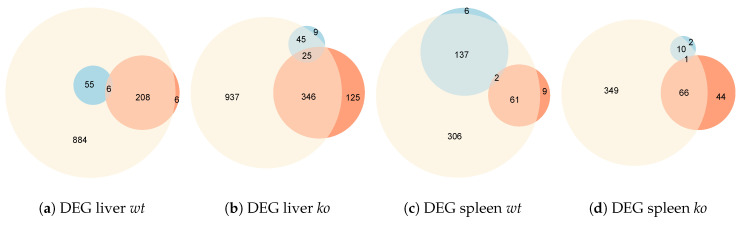
Euler diagrams showing the overlapping of DEG between the three possible contrast situations: control vs. cases at 3 d p.i. (red), control vs. cases at 5 d p.i. (yellow) and cases at 3 d p.i. vs. cases at 5 d p.i. (blue) *ko* refers to *Ly6E^*Δ*HSC^* samples. These comparisons were performed both organ and genotype-wise considering four groups of samples: (**a**) liver *wt*, (**b**) liver *Ly6E^*Δ*HSC^*, (**c**) spleen *wt*, (**d**) spleen *Ly6E^*Δ*HSC^*.

For both genotypes and organs, the results of the differential expression analyses reveal that MHV injection triggers a progressive genetic program from the control situation to the MHV-infected scenario at 5 d p.i., as shown in Figure 3a. The absolute number of DEG between control vs. cases at 5 d p.i. was considerably larger than in the comparison between control vs. cases at 3 d p.i. Furthermore, in all cases, most of the DEG in control vs. cases at 3 d p.i. are also differentially-expressed in the control vs. cases at 5 d p.i. comparison, as shown in Figure 4.

**Figure A3:**
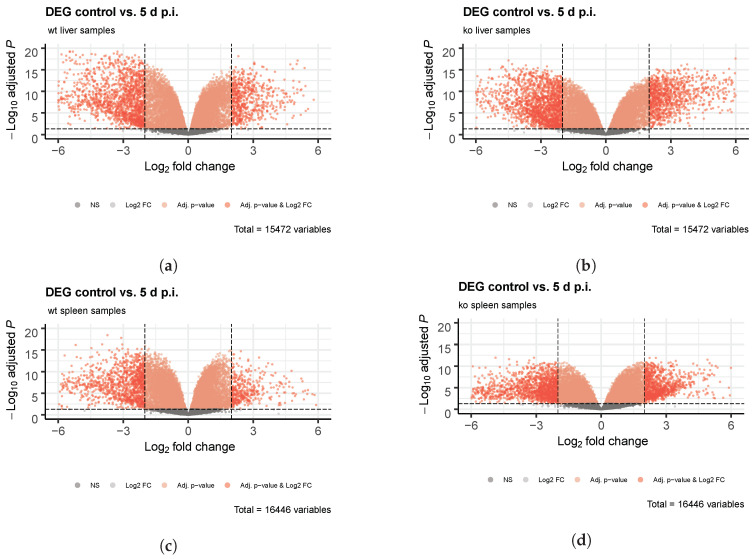
Volcano plots showing the differentially-expressed genes (DEG) that proceeded to the analyses. DEG were filtered by log_2_FC ≥ 2 and adjusted *p* value ≤ 0.05. These comparisons were performed both organ and genotype-wise: (**a**) liver *wt*, (**b**) liver *ko*, (**c**) spleen *wt*, (**d**) spleen *ko*. *ko*, *Ly^*Δ*6EDHSC^*

**Figure A4:**
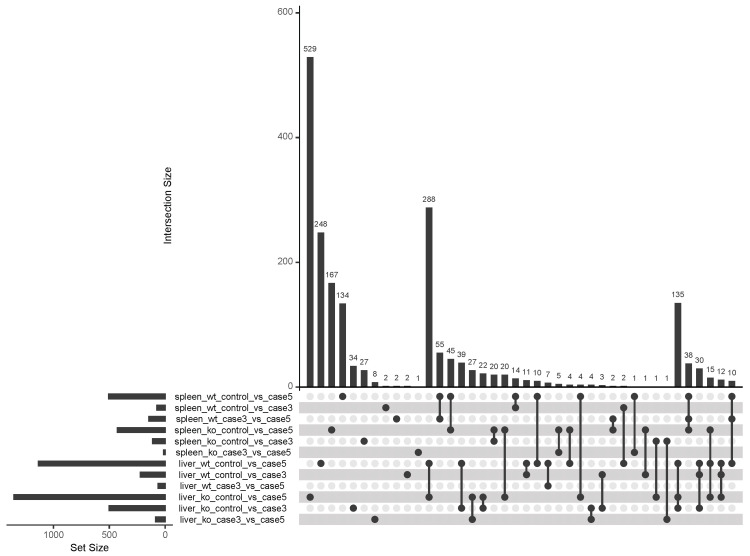
UpSet plot representing the commonalities between the 12 differentially-expressed genes (DEG) groups identified in differential expression analyses. The comparison of controls vs. samples at 5 d p.i. comprised the greatest number of genes for all sample types.

Regarding genes fold change, an overall genetic up-regulation is observed upon infection. Around 70% of DEG are upregulated for all the comparisons performed for *wt* samples, as shown in Figure 3b. Nonetheless, a dramatic reduce in this genetic up-regulation is observed, by contrast, in *knockout* samples, even limiting upregulated genes to nearly 50% in the control vs. cases at 3 d p.i. comparison of liver *Ly6E*${}^{\Delta HSC}$ samples. The largest differences are observed in the comparison of controls vs. cases at 5 d p.i (Figure A3 and Figure A4). These DEG are of great interest for the understanding of the immune response of both *wt* and *ko* mice to viral infection. These genes were selected to filter the original dataset for latter network reconstruction.

The commonalities between *wt* and *ko* control samples for both organs were also verified through differential expression analysis following the same criteria (Log2FC > 2, *p* value < 0.05). The number of DEG between *wt* and *ko* liver control samples (2) and between *wt* and *ko* spleen control samples (20) were not considered significant, so samples were taken as analogous starting points for infection.

### 4.3. Reconstruction and Analysis of Gene Networks

As stated above, the samples were arranged both organ and genotype-wise in order to generate networks which would model the progress of the disease in each scenario. GCNs were inferred from Log2-normalized expression datasets. A count of 1 was added at log2 normalization so the problem with remaining zero values was avoided. Each network was generated exclusively taking into consideration their corresponding DEG at control vs. cases at 5 d p.i., where larger differences were observed. Four networks were then reconstructed from these previously-identified DEG for liver *wt* samples (1133 genes), liver *ko* samples (1153 genes), spleen *wt* samples (506 genes) and spleen *ko* samples (426 genes). This approach results in the modeling of only those relationships that are related to the viral infection. Each sample set was then fed to *EnGNet* for the reconstruction of the subsequent network. Genes that remained unconnected due to weak relationships, which do not overcome the set threshold, were removed from the networks. Furthermore, the goodness of *EnGNet*-generated models outperformed other well-known inference approaches, as detailed in Appendix B.

**Figure A5:**
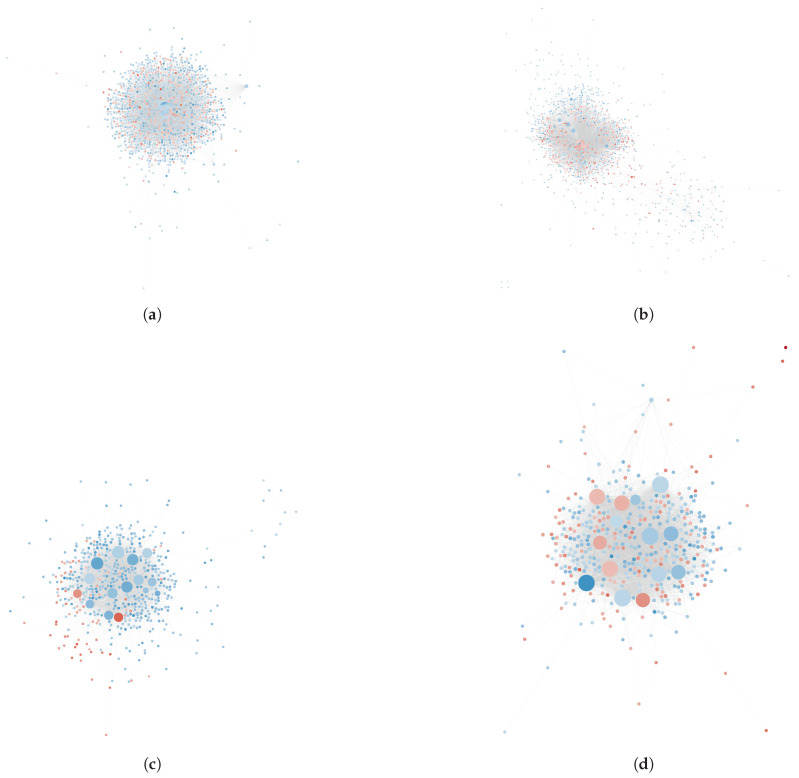
Inferred networks for (**a**) liver *wt* (1118 nodes, 16,281 edges, 4 clusters), (**b**) liver *ko* (1300 nodes, 15,727 edges, 3 clusters), (**c**) spleen *wt* (485 nodes, 4042 edges, 3 clusters), (**d**) spleen *ko* (403 nodes, 4220 edges, 4 clusters). Nodes are colored according to log2FC, upregulated genes in blue, downregulated genes in red. Clusters are numbered from left to right. Node size is represented according to node’s degree. Edge transparency is represented according to edge weight. Networks are displayed using the yfiles organic layout [92].

Topological parameters were estimated and added as node attributes using *igraph*, together with Log2FC, prior to Cytoscape import. Specifically, networks were simplified by removing potential loops and multiple edges. The clustering topological scrutiny of the reconstructed networks revealed neat modules in all cases, as shown in Figure A5. The number of clusters identified in each network, as well as the number of genes harbored in the clusters is shown in Table A1.

**Figure 5:**
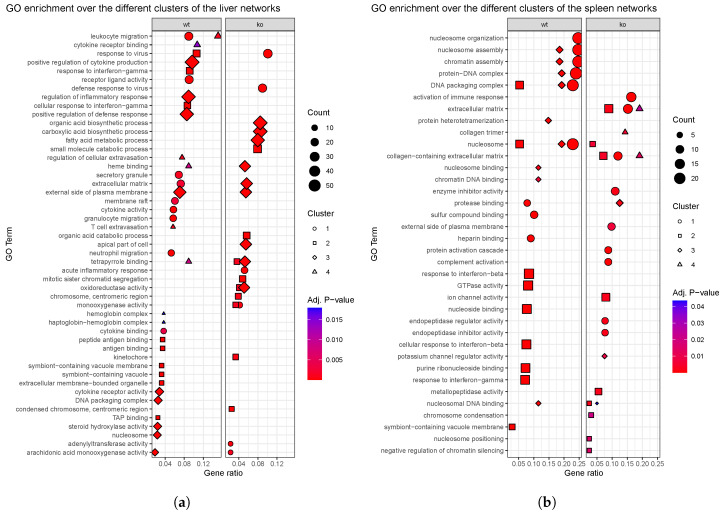
Enrichment analyses performed over the main clusters identified in *wt* and *ko* networks of (**a**) liver and (**b**) spleen networks. Gene ratio is defined by the number of genes used as input for the ernichment analyses associated with a particular GO term divided by the total number of input genes.

As already mentioned, according to gene networks theory, nodes contained within the same cluster are often involved in the same biological process [5,81]. In this context, the GO-based enrichment analyses over the identified clusters may well provide an idea of the affected functions. Only clusters containing more than 10 genes were considered, since this is the minimum number of elements required by the enrichment tool *ClusterProfiler*. The results of the enrichment analyses revealed that most GO terms were not shared between *wt* and *ko* homologous samples, as shown in Figure 5.

**Figure A6:**
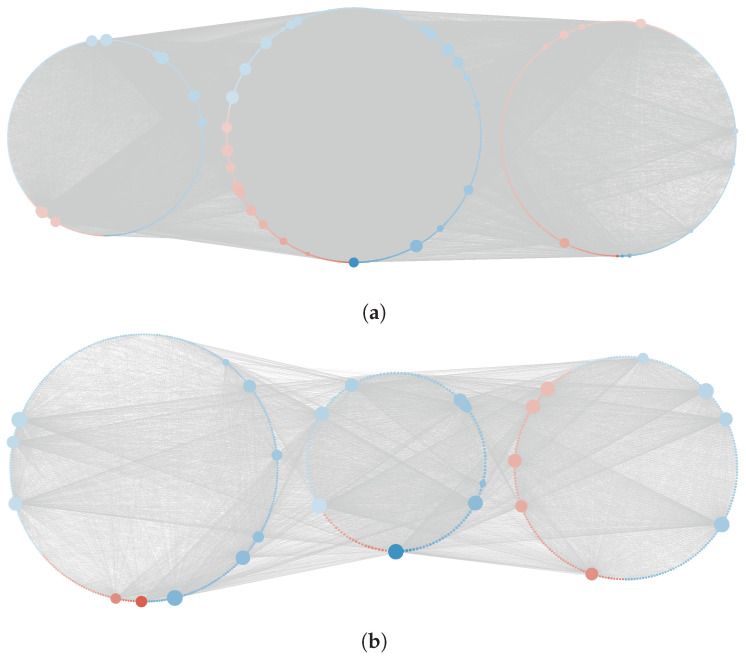
Networks resulting from the organ-wise merging of (**a**) *wt* and (**b**) *ko* samples. From left to right, nodes are displayed in circles depending on whether genes are contained exclusively at the *wt*, in the intersection between the *ko* and *wt* networks and in the *ko* network exclusively. Nodes are sorted and colored according to log2FC, upregulated genes in blue, downregulated genes in red. Node size is represented according to node’s degree.

**Figure A7:**
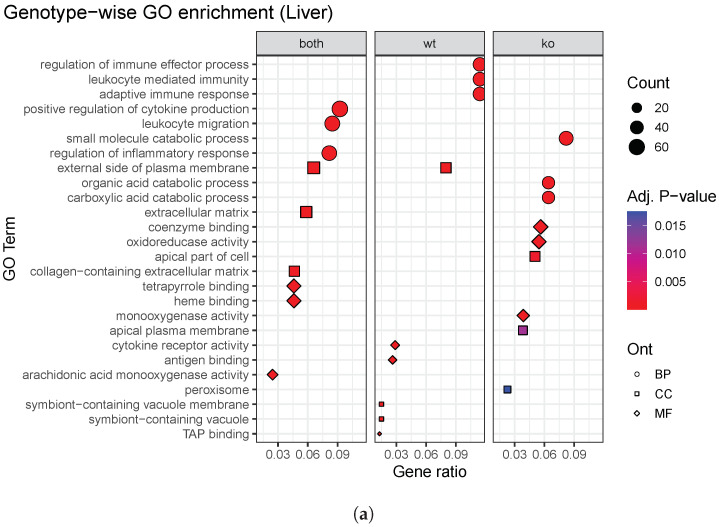

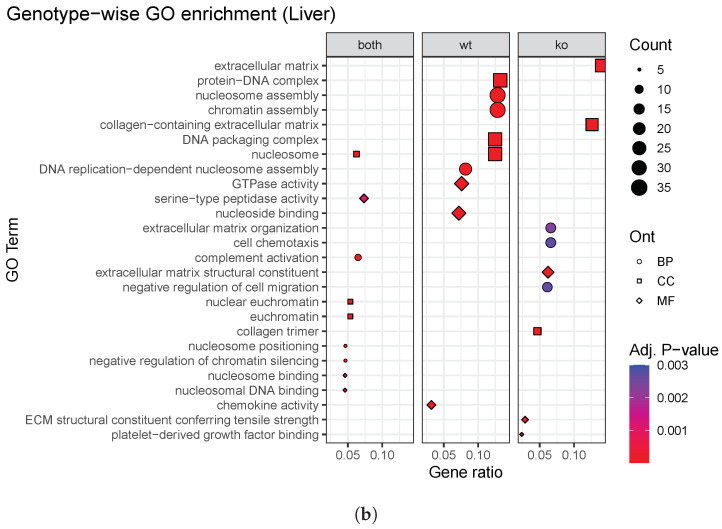
Enrichment analyses based on node exclusiveness of (**a**) liver and (**b**) spleen networks. *wt* refers to nodes exclusively present at those networks reconstructed from *wt* samples; *ko* refers to nodes exclusively present at networks reconstructed from *Ly6E*${}^{\Delta HSC}$ samples; *both* addresses shared nodes between *wt* and *ko* networks. Gene ratio is defined by the number of genes used as input for the ernichment analyses associated with a particular GO term divided by the total number of input genes.

In order to further explore the reconstructed networks, the intersection of *ko* and *wt* networks of a same organ was computed. This refers to the genes and relationships that are shared between both genotypes for a specific organ. Additionally, the genes and relationships that were exclusively present at the *wt* and *ko* samples were also estimated, as shown in Figure A6. The enrichment analyses over the nodes, separated using this criterion, would reveal the biological processes that make the difference between in *Ly6E*${}^{\Delta HSC}$ mice compared to *wt* ones. The results of such analyses are shown in Figure A7.

**Figure 6:**
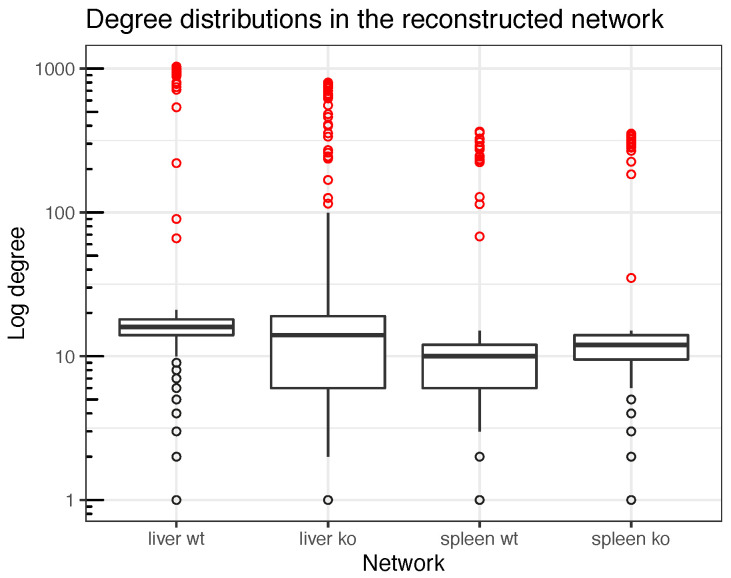
Boxplots representative of the degree distributions for each one of the four reconstructed networks. Identified hubs, according to the Q3+1.5×IQR criterion, are highlighted in red. The degree cutoffs, above which nodes would be considered as hubs, were 24, 39, 21 and 21, respectively for liver *wt*, liver *ko*, spleen *wt* and spleen *ko* networks. Note degree is represented in a log scale given that the reconstructed networks present a scale-free topology.

**Figure A8:**
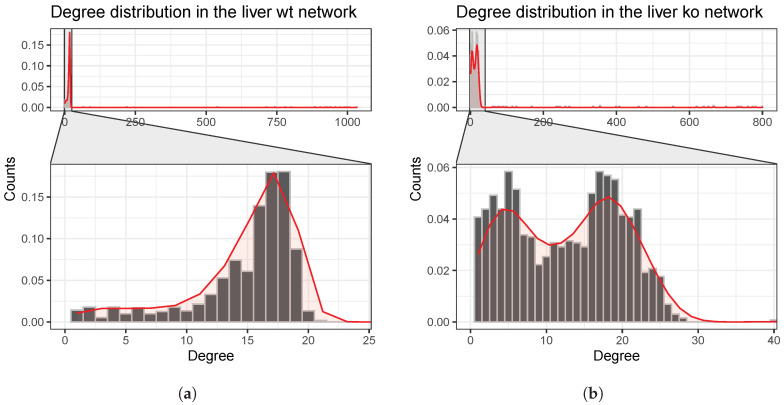

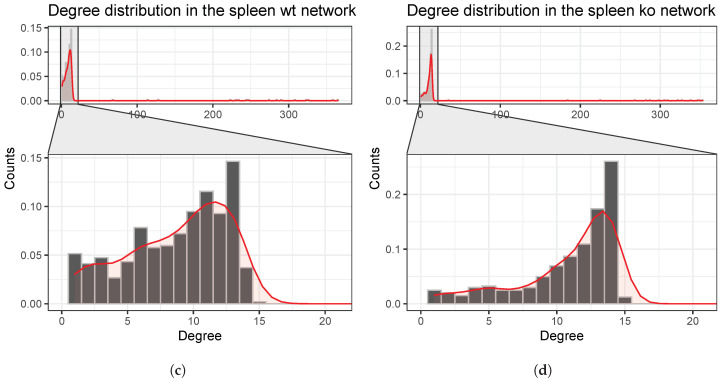
Distribution of node’s degree throughout the networks reconstructed from (**a**) liver *wt* samples, (**b**) liver *ko* samples, (**c**) spleen *wt* samples and (**d**) spleen *ko* samples. The distribution trendline is shown in red. Nodes that are not present in the zoomed area are considered hubs. Note degree distributions do not fit a normal distribution (Shapiro–Wilk normality test, *p*-value < 0.05)。

Finally, the exploration of nodes’ *degree* distribution would reveal those genes that can be considered hubs. Those nodes comprised within the top genes with highest degree (degree > *Q*3 + 1.5 × *IQ*), also known as upper outliers in the nodes distribution, were considered hubs. A representation of nodes’ degree distribution throughout the four reconstructed networks is shown in Figure 6. These distributions are detailed in Figure A8. This method provided four cutoff values for the degree, 24, 39, 21 and 21, respectively for liver *wt* and *ko*, spleen *wt* and *ko* networks. Above these thresholds, nodes would be considered as hubs in each network. These hubs are shown in Table A2,Table A3,Table A4 and Table A5.

## 5. Discussion

In this work four gene networks were reconstructed to model the genetic response MHV infection in two tissues, liver and spleen, and in two different genetic backgrounds, *wild type* and *Ly6E*${}^{\Delta HSC}$. Samples were initially explored in order to design an inference rationale. Not only did the designed approach reveal major differences between the genetic programs in each organ, but also, between different subgroups of samples, in a time-series-like manner. Noticeably, disparities between *wt* and *Ly6E*${}^{\Delta HSC}$ samples were observed in both tissues, and differential expression analyses revealed relevant differences in terms of the immune response generated. Hereby, our results predict the impact of *Ly6E ko* on HSC, which resulted in an impaired immune response compared to the *wt* situation.

### 5.1. Exploratory Analyses Revealed a Time-Series Llike Behaviour on Raw Data, Assisting Network Reconstruction

Overall, results indicate that the reconstruction rationale, elucidated from exploratory findings, is suitable for the modeling of the viral progression. Regarding the variance in gene expression in response to virus, PCA and K-medoid clustering revealed strong differences between samples corresponding to liver spleen, respectively (Figure 2a). These differences set the starting point for the modeling approach, in which samples corresponding to each organ were analyzed independently. This *modus operandi* is strongly supported by the tropism that viruses exhibit for certain tissues, which ultimately results in a differential viral incidence and charge depending on the organ [82]. In particular, the liver is the target organ of MHV, identified as the main disease site [83]. On the other hand, the role of the spleen in innate and adaptive immunity against MHV has been widely addressed [84,85]. The organization of this organ allows blood filtration for the presentation of antigens to cognate lymphocytes by the antigen presenting cells (APCs), which mediate the immune response exerted by T and B cells [86].

As stated before, PCA revealed differences between the three sample groups on each organ: control and MHV-infected at 3 and 5 d p.i. Interestingly, between-groups differences are specially clear for liver samples (Figure 2b), whereas spleen samples are displayed in a continuum-like way. This becomes more evident in organ-wise PCA (Figure 2), and was latter confirmed by the exploration of the top 500 most variable genes and differential expression analyses (Figure A2). Furthermore, clear differences between *wt* and *Ly6E*${}^{\Delta HSC}$ samples are observed in none of these analyses, although the examination of the differential expression and network reconstruction did exposed divergent immune responses for both genotypes.

### 5.2. Differential Expression Analyses Revealed Significant Changes between Wild Type and Knockout Samples

The differential expression analyses revealed the progressive genetic response to virus for both organs and genotypes (Figure 3a and Figure 4). In a *wt* genetic background, MHV infection causes an overall rise in the expression level of certain genes, as most DEG in cases vs. control samples are upregulated. However, in a *Ly6E*${}^{\Delta HSC}$ genetic background, this upregulation is not as prominent as in a *wt* background, significantly reducing the number of upregulated genes (Figure 3b). Besides, the number of DEG in each comparison varies from *wt* to *Ly6E*${}^{\Delta HSC}$ samples.

Attending at the DEG in the performed comparisons, for both the *wt* and *ko* genotypes, liver cases at 3 d p.i. are more similar to liver cases at 5 d p.i. than to liver controls, since the number of DEG between the first two measuring points is significantly lower than the number of DEG between control and case samples at 3 d p.i. (Figure 4a,b). A different situation occurs in the spleen, where *wt* cases at 3 d p.i. are closer to control samples (Figure 4c), whereas *ko* cases at 3 d p.i. seem to be more related to cases at 5 d p.i. (Figure 4d). This was already suggested by hierarchical clustering in the analysis of the top 500 most variable genes, and could be indicative of a different progression of the infection impact on both organs, which could be modulated by gene *Ly6E*, at least for the spleen samples.

**Figure A9:**
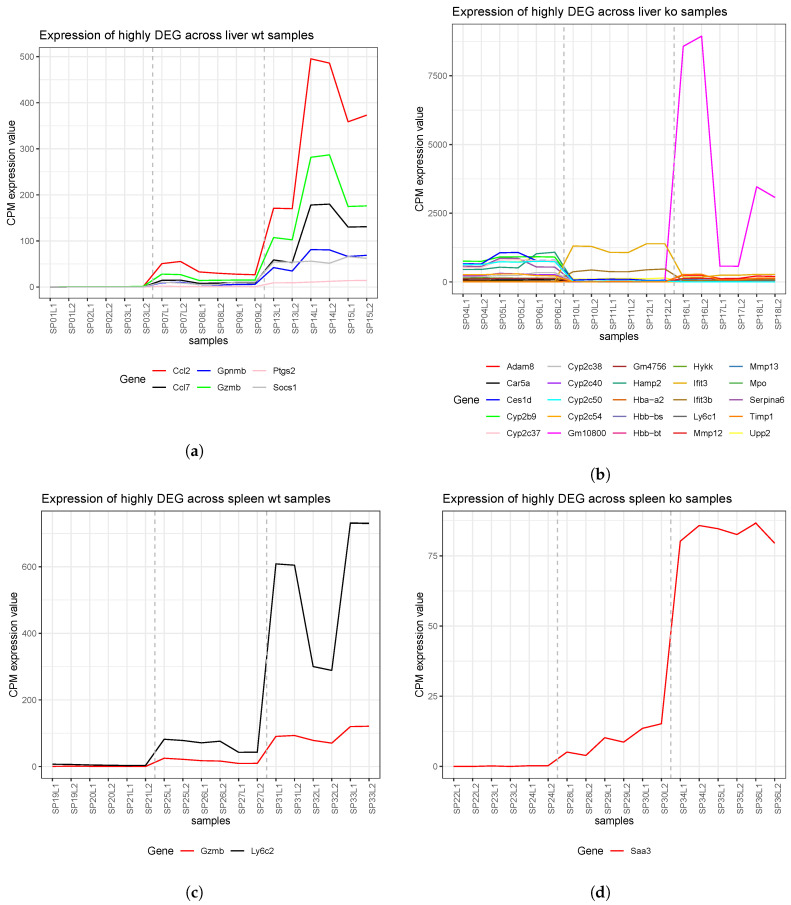
CPM-normalized expression values of highly DEG identified across (**a**) liver *wt* samples, (**b**) liver *ko* samples, (**c**) spleen *wt* samples and (**d**) spleen *ko* samples. Dashed lines separate samples from the three groups under study: controls, cases at 3 d p.i. and cases at 5 d p.i. Note sample order within same group is exchangeable.

Moreover, the results of the DEG analyses indicate that the sole *knockout* of gene *Ly6E* in HSC considerably affects the upregulating genetic program normally triggered by viral infection in *wild type* individuals (in both liver and spleen). Interestingly, there are some genes in each organ and genotype that are differentially expressed in every comparison between the possible three sample types, controls, cases at 3 d p.i. and cases at 5 d p.i. These genes, which we termed highly DEG, could be linked to the progression of the infection, as changes in their expression level occur with days post injection, according to the data. The rest of the DEG, show an uprise or fall when comparing two sample types, which does not change significantly in the third sample type. Alternatively, highly DEG, shown in Table A6, exhibited three different expression patterns: (i) Their expression level, initially low, rises from control to cases at 3 d p.i. and then rises again in cases at 5 d p.i. (ii) Their expression level, initially high in control samples, falls at 3 d p.i. and falls even more at 5 d p.i cases. (iii) Their expression level, initially low, rises from control to cases at 3 d p.i. but then falls at cases at 5 d p.i., when it is still higher than the initial expression level. These expression patterns, which are shown in Figure A9, might be used to keep track of the disease progression, differentiating early from late infection stages.

In some cases, these genes exhibited inconsistent expression levels, specially at 5 d p.i. cases, which indicates the need for further experimental designs targeting these genes. Highly DEG could be correlated with the progression of the disease, as in regulation types (i) and (ii) or by contrast, be required exclusively at initial stages, as in regulation type (iii). Notably, genes *Gm10800* and *Gm4756* are predicted genes which, to date, have been poorly described. According to the *STRING* database [79], *Gm10800* is associated with gene *Lst1* (Leukocyte-specific transcript 1 protein), which has a possible role in modulating immune responses. In fact, *Gm10800* is homologous to human gene PIRO (Progranulin-Induced-Receptor-like gene during Osteoclastogenesis), related to bone homeostasis [87,88]. Thus, we hypothesize that bone marrow-derived cell lines, including erythrocytes and leukocytes (immunity effectors), could also be regulated by *Gm10800*. On the other hand, *Gm4756* is not associated to any other gene according to *STRING*. Protein *Gm4756* is homologous to Human protein DHRS7 (dehydrogenase/reductase SDR family member 7) isoform 1 precursor. Nonetheless and to the best of our knowledge, these genes have not been previously related to *Ly6E*, and could play a role in the immune processes mediated by this gene.

Finally, highly DEG were not found exclusively present in *wt* nor *ko* networks, instead, these were common nodes of these networks for each organ. This suggests that highly DEG might be of core relevance upon MHV infection, with a role in those processes independent on *Ly6E*${}^{\Delta HSC}$. Besides, genes *Hykk*, *Ifit3* and *Ifit3b*; identified as highly DEG throughout liver *Ly6E*${}^{\Delta HSC}$ samples were also identified as hubs in the liver *ko* network. Also gene *Saa3*, highly DEG across spleen *Ly6E*${}^{\Delta HSC}$ samples was considered a hub in the spleen *ko* network. Nevertheless, these highly DEG require further experimental validation.

### 5.3. The Ablation of *Ly6E* in HSC Results in Impaired Immune Response as Predicted by Enrichment Analyses

The enrichment analyses of the identified clusters at each network revealed that most GO terms are not shared between the two genotypes (Figure 5), despite the considerable amount of shared genes between the two genotypes for a same organ. The network reconstructed from liver *wt* samples reflects a strong response to viral infection, involving leukocyte migration or cytokine and interferon signaling among others. These processes, much related to immune processes, are not observed in its *ko* counterpart.

The liver *wt* network presented four clusters (Figure A5a). Its cluster 1 regulates processes related to leukocyte migration, showing the implication of receptor ligand activity and cytokine signaling, which possibly mediates the migration of the involved cells. Cluster 2 is related to interferon-gamma for the response to MHV, whereas cluster 3 is probably involved in the inflammatory response mediated by pro-inflammatory cytokines. Last, cluster 4 is related to cell extravasation, or the leave of blood cells from blood vessels, with the participation of gene *Nipal1*. The positive regulation observed across all clusters suggests the activation of these processes. Overall, hub genes in this network have been related to the immune response to viral infection, as the innate immune response to the virus is the mediated by interferons. Meanwhile, the liver *ko* network showed three main clusters (Figure A5b). Its cluster 1 would also be involved in defense response to virus, but other processes observed in the liver *wt* network, like leukocyte migration or cytokine activity, are not observed in this cluster nor the others. Cluster 2 is then related to the catabolism of small molecules and cluster 3 is involved in acids biosynthesis. These processes are certainly ambiguous and do not correspond the immune response observed in the *wt* situation, which suggests a decrease in the immune response to MHV as a result of *Ly6E* ablation in HSC.

On the other hand, spleen *wt* samples revealed high nuclear activity potentially involving nucleosome remodeling complexes and changes in DNA accessibility. Histone modification is a type of epigenetic modulation which regulates gene expression. Taking into account the central role of the spleen in the development of immune responses, the manifested relevance of chromatin organization could be accompanied by changes in the accessibility of certain DNA regions with implications in the spleen-dependent immune response. This is supported by the reduced reaction capacity in the first days post-infection of *Ly6E*${}^{\Delta HSC}$ samples compared to *wt*, as indicated by the number of DEG between control and cases at 3 d p.i for these genotypes. The spleen *wt* network displayed three clusters (Figure A5c). Cluster 1, whose genes were all upregulated in *Ly6E*${}^{\Delta HSC}$ samples at 5 d p.i. compared to mock infection, is mostly involved in nucleosome organization and chromatin remodelling, together with cluster 3. Cluster 2 would also be related to DNA packaging complexes, possibly in response to interferon, similarly to liver networks. Instead, in spleen *ko* most genes take part in processes related to the extracellular matrix. In the spleen *ko* network, four clusters were identified (Figure A5d). Cluster 1 is related to the activation of an immune response, but also, alongside with clusters 2 and 4, to the extracellular matrix, possibly in relation with collagen, highlighting its role in the response to MHV. Cluster 3 is implied in protease binding. The dramatic shut down in the *ko* network of the nuclear activity observed in the spleen *wt* network, leads to the hypothesis that the chromatin remodeling activity observed could be related to the activation of certain immunoenhancer genes, modulated by gene *Ly6E*. In any case, further experimental validation of these results would provide meaningful insights in the face of potential therapeutic approaches (See Appendix A for more details).

The exploration of nodes memebership, depending on whether these exclusively belonged to *wt* or *ko* networks or, by contrast, were present in both networks, helped to understand the impairment caused by *Ly6E*${}^{\Delta HSC}$. In this sense, GO enrichment analyses over these three defined categories of the nodes in the liver networks revealed that genes at their intersection are mainly related to cytokine production, leukocyte migration and inflammatory response regulation, in accordance to the phenotype described for MHV-infection [89]. However, a differential response to virus is observed in *wt* mice compared to *Ly6E*-ablated. The nodes exclusively present at the *wt* liver network are related to processes like regulation of immune effector process, leukocyte mediated immunity or adaptive immune response. These processes, which are found at a relatively high gene ratio, are not represented by nodes exclusively present in the liver *ko* network. Additionally, genes exclusively present at the *wt* network and the intersection network are upregulated in case samples with respect to controls (Figure A6a), which suggests the activation of the previously mentioned biological processes. On the other hand, genes exclusively-present at the liver *ko* networks, mostly down-regulated, were found to be associated with catabolism.

As for the spleen networks, genotype-wise GO enrichment results revealed that the previously-mentioned intense nuclear activity involving protein-DNA complexes and nucleosome assembly is mostly due to *wt*-exclusive genes. Actually, these biological processes could be pinpointing cell replication events. Analogously to the liver case, genes that were found exclusively present in the *wt* network and the intersection network are mostly upregulated, whereas in the case of *ko*-exclusive genes the upregulation is not that extensive. Interestingly, the latter are mostly related to extracellular matrix (ECM) organization, which suggest the relevance of *Ly6E* on these. Other lymphocyte antigen-6 (LY-6) superfamily members have been related to ECM remodelling processes such as the Urokinase receptor (*uPAR*), which participates in the proteolysis of ECM proteins [90]. However and to the best of our knowledge, the implications of *Ly6E* in ECM have not been reported.

The results presented are in the main consistent with those by Pfaender et al. [54], who observed a loss of genes associated with the type I IFN response, inflammation, antigen presentation, and B cells in infected *Ly6E*${}^{\Delta HSC}$ mice. Genes *Stat1* and *Ifit3*, selected in their work for their high variation in absence of *Ly6e*, were identified as hub genes in the networks reconstructed from liver *wild type* and *knockout* samples, respectively. It is to be noticed that our approach significantly differs to the one carried out in the original study. In this particular case, we consider that the reconstruction of GCN enables a more comprehensive analysis of the data, potentially finding the key genes involved in the immune response onset and their relationships with other genes. For instance, the transcriptomic differences between liver and spleen upon *Ly6E* ablation become more evident using GCN.

Altogether, the presented results show the relevance of gene *Ly6E* in the immune response against the infection caused by MHV. The disruption of *Ly6E* significantly reduced the immunogenic response, affecting signaling and cell effectors. These results, combining *in vivo* and *in silico* approaches, deepen in our understanding of the immune response to viruses at the gene level, which could ultimately assist the development of new therapeutics. For example, basing on these results, prospective studies on *Ly6E* agonist therapies could be inspired, with the purpose of enhancing the gene expression level via gene delivery. Given the relevance of *Ly6E* in SARS-CoV-2 according to previous studies [54,91], the overall effects of *Ly6E* ablation in HSCs upon SARS-CoV-2 infection, putting special interest in lung tissue, might show similarities with the deficient immune response observed in the present work.

## 6. Conclusions

In this work we have presented an application of co-expression gene networks to analyze the global effects of *Ly6E* ablation in the immune response to MHV coronavirus infection. To do so, the progression of the MHV infection on the genetic level was evaluated in two genetic backgrounds: wild type mice (*wt*, Ly6Efl/fl) and Ly6E *knockout* mutants (*ko*, *Ly6E^*Δ*HSC^*) mice. For these, viral progression was assessed in two different organs, liver and spleen.

The proposed reconstruction rationale revealed significant differences between MHV-infected *wt* and *Ly6E*${}^{\Delta HSC}$ mice for both organs. In addition we observed that MHV infection triggers a progressive genetic response of upregulating nature in both liver and spleen. In addition, the results suggest that the ablation of gene *Ly6E* at HSC caused an impaired genetic response in both organs compared to *wt* mice. The impact of such ablation is more evident in the liver, consistently with the disease site. At the same time, the immune response in the spleen, which seemed to be mediated by an intense chromatin activity in the normal situation, is replaced by ECM remodeling in *Ly6E*${}^{\Delta HSC}$ mice.

We infer that the presence of *Ly6E* limits the damage in the above mentioned target sites. We believe that the characterization of these processes could motivate the efforts towards novel antiviral approaches. Finally, in the light of previous works, we hypothesize that *Ly6E* ablation might show analogous detrimental effects on immunity upon the infection caused by other viruses including SARS-CoV, MERS and SARS-CoV-2. In future works, we plan to investigate whether the over-expression of *Ly6E* in *wt* mice has an enhancement effect in immunity. In this direction, *Ly6E* gene mimicking (agonist) therapies could represent a promising approach in the development of new antivirals.

## Figures and Tables

**Figure A10:**
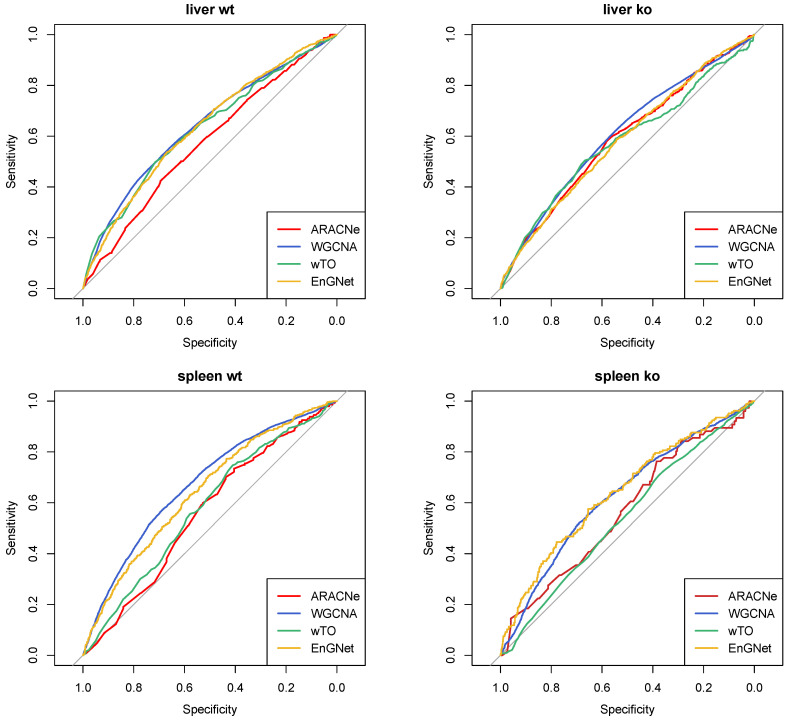
Receiver operating characteristic (ROC) curves for the four datasets obtained in our study using different reconstruction methods. Sensitivity is the true positive rate: TP/(TP+FN). Specificity is the true negative rate: TN/(TN+FP). TP, true positive; TN, true negative; FN, false negative; FP, false positive.

**Figure A11:**
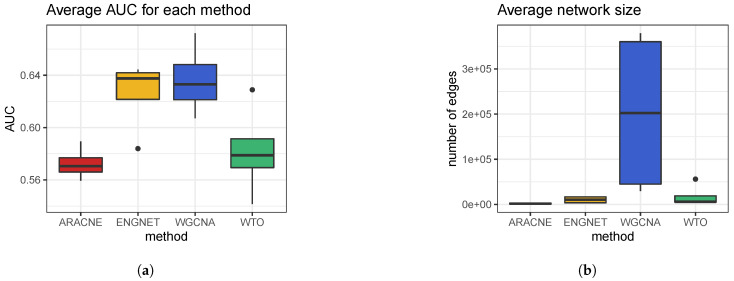
(**a**) Comparison of the average area under the ROC curve (AUC) for the four reconstruction methods under comparison across the four used datasets. On average, EnGNet outperformed the other three methods in terms of AUC. (**b**) Size comparison of the inferred networks. EnGNet exhibited competitive results in terms of network size, providing considerably sparser networks than WGCNA’s

## Appendix A. Figures and Tables

**Table A1:** Number of DEG used as input to EnGNet for network reconstruction and their latter distribution in inferred networks. Genes that were not assigned to a cluster (or were comprised in minoritary clusters) were not taken into consideration for enrichment analyses.

	Liver *wt*	Liver *ko*	Spleen *wt*	Spleen *ko*
Input genes	1133	1153	506	426
Network genes	1118	1300	485	403
Cluster 1	262	284	180	109
Cluster 2	218	379	255	190
Cluster 3	579	624	36	77
Cluster 4	59			25
Unconnected/minor clustered	0	13	14	2

**Table A2:** Hubs identified in the network reconstructed from liver *wt* samples. Degree cutoff: 24. Reg. regulation.

Ensembl ID	Cluster	Degree	Reg.	Symbol	Description
ENSMUSG00000034593	1	1033	up	Myo5a	myosin VA
ENSMUSG00000000982	3	1006	up	Ccl3	chemokine (C-C motif) ligand 3
ENSMUSG00000030745	2	997	up	Il21r	interleukin 21 receptor
ENSMUSG00000032322	3	989	up	Pstpip1	proline-serine-threonine phosphatase-interacting protein 1
ENSMUSG00000079227	3	975	up	Ccr5	chemokine (C-C motif) receptor 5
ENSMUSG00000031304	3	957	up	Il2rg	interleukin 2 receptor, gamma chain
ENSMUSG00000069268	3	940	up	Hist1h2bf	histone cluster 1, H2bf
ENSMUSG00000027071	1	938	down	P2rx3	purinergic receptor P2X, ligand-gated ion channel, 3
ENSMUSG00000019232	3	929	down	Etnppl	ethanolamine phosphate phospholyase
ENSMUSG00000032643	3	921	up	Fhl3	four and a half LIM domains 3
ENSMUSG00000033763	3	904	down	Mtss2	MTSS I-BAR domain containing 2
ENSMUSG00000032094	1	887	up	Cd3d	CD3 antigen, delta polypeptide
ENSMUSG00000050896	3	883	up	Rtn4rl2	reticulon 4 receptor-like 2
ENSMUSG00000067219	4	801	down	Nipal1	NIPA-like domain containing 1
ENSMUSG00000110439	3	780	down	Mup22	major urinary protein 22
ENSMUSG00000004105	2	743	down	Angptl2	angiopoietin-like 2
ENSMUSG00000081650	1	713	up	Gm16181	-
ENSMUSG00000050395	2	538	up	Tnfsf15	tumor necrosis factor (ligand) superfamily, member 15
ENSMUSG00000038067	1	220	up	Csf3	colony stimulating factor 3 (granulocyte)
ENSMUSG00000026104	2	90	up	Stat1	signal transducer and activator of transcription 1
ENSMUSG00000037965	2	66	up	Zc3h7a	zinc finger CCCH type containing 7 A

**Table A3:** Hubs identified in the network reconstructed from liver *Ly6E*${}^{\Delta HSC}$ samples. Degree cutoff: 39. Reg. regulation.

Ensembl ID	Cluster	Degree	Reg.	Symbol	Description
ENSMUSG00000029445	2	800	down	Hpd	4-hydroxyphenylpyruvic acid dioxygenase
ENSMUSG00000037071	3	781	down	Scd1	stearoyl-Coenzyme A desaturase 1
ENSMUSG00000041773	3	773	up	Enc1	ectodermal-neural cortex 1
ENSMUSG00000075015	3	760	up	Gm10801	-
ENSMUSG00000021250	3	742	up	Fos	FBJ osteosarcoma oncogene
ENSMUSG00000031618	3	735	down	Nr3c2	nuclear receptor subfamily 3, group C, member 2
ENSMUSG00000022419	1	732	down	Deptor	DEP domain containing MTOR-interacting protein
ENSMUSG00000033610	3	700	down	Pank1	pantothenate kinase 1
ENSMUSG00000024349	3	667	up	Tmem173	transmembrane protein 173
ENSMUSG00000006519	3	666	up	Cyba	cytochrome b-245, alpha polypeptide
ENSMUSG00000035878	3	666	down	Hykk	hydroxylysine kinase 1
ENSMUSG00000054630	2	652	down	Ugt2b5	UDP glucuronosyltransferase 2 family, polypeptide B5
ENSMUSG00000041757	3	639	down	Plekha6	pleckstrin homology domain containing, family A member 6
ENSMUSG00000053398	3	620	up	Phgdh	3-phosphoglycerate dehydrogenase
ENSMUSG00000022025	3	555	down	Cnmd	chondromodulin
ENSMUSG00000029659	2	482	up	Medag	mesenteric estrogen dependent adipogenesis
ENSMUSG00000062380	2	461	up	Tubb3	tubulin, beta 3 class III
ENSMUSG00000069309	3	408	up	Hist1h2an	histone cluster 1, H2an
ENSMUSG00000034285	3	399	down	Nipsnap1	nipsnap homolog 1
ENSMUSG00000027654	3	355	up	Fam83d	family with sequence similarity 83, member D
ENSMUSG00000073435	2	355	down	Nme3	NME/NM23 nucleoside diphosphate kinase 3
ENSMUSG00000021062	2	336	up	Rab15	RAB15, member RAS oncogene family
ENSMUSG00000037852	3	271	up	Cpe	carboxypeptidase E
ENSMUSG00000096201	2	260	up	Gm10715	-
ENSMUSG00000022754	2	245	up	Tmem45a	transmembrane protein 45a
ENSMUSG00000038233	1	239	down	Gask1a	golgi associated kinase 1A
ENSMUSG00000043456	2	236	up	Zfp536	zinc finger protein 536
ENSMUSG00000095891	2	168	up	Gm10717	-
ENSMUSG00000096688	1	126	down	Mup17	major urinary protein 17
ENSMUSG00000099398	2	115	up	Ms4a14	membrane-spanning 4-domains, subfamily A, member 14
ENSMUSG00000025002	1	99	down	Cyp2c55	cytochrome P450, family 2, subfamily c, polypeptide 55
ENSMUSG00000074896	1	91	up	Ifit3	interferon-induced protein with tetratricopeptide repeats 3
ENSMUSG00000062488	1	86	up	Ifit3b	interferon-induced protein with tetratricopeptide repeats 3B
ENSMUSG00000029417	1	78	up	Cxcl9	chemokine (C-X-C motif) ligand 9
ENSMUSG00000057465	1	77	up	Saa2	serum amyloid A 2
ENSMUSG00000050908	2	69	up	Tvp23a	trans-golgi network vesicle protein 23A
ENSMUSG00000030142	1	63	up	Clec4e	C-type lectin domain family 4, member e
ENSMUSG00000038751	1	61	down	Ptk6	PTK6 protein tyrosine kinase 6
ENSMUSG00000068606	1	40	up	Gm4841	predicted gene 4841

**Table A4:** Hubs identified in the network reconstructed from spleen *wt* samples. Degree cutoff: 21. Reg. regulation.

Ensembl ID	Cluster	Degree	Reg.	Symbol	Description
ENSMUSG00000019505	2	365	up	Ubb	ubiquitin B
ENSMUSG00000094777	2	358	up	Hist1h2ap	histone cluster 1, H2ap
ENSMUSG00000057729	3	326	up	Prtn3	proteinase 3
ENSMUSG00000056071	1	323	up	S100a9	S100 calcium binding protein A9 (calgranulin B)
ENSMUSG00000025403	2	308	up	Shmt2	serine hydroxymethyltransferase 2 (mitochondrial)
ENSMUSG00000023132	2	290	up	Gzma	granzyme A
ENSMUSG00000078920	2	284	up	Ifi47	interferon gamma inducible protein 47
ENSMUSG00000037894	1	274	up	H2afz	H2A histone family, member Z
ENSMUSG00000035472	2	247	down	Slc25a21	solute carrier family 25 (mitochondrial oxodicarboxylate carrier), member 21
ENSMUSG00000009350	1	244	up	Mpo	myeloperoxidase
ENSMUSG00000103254	1	234	up	Ighv1-15	-
ENSMUSG00000069274	1	230	up	Hist1h4f	histone cluster 1, H4f
ENSMUSG00000028328	2	223	down	Tmod1	tropomodulin 1
ENSMUSG00000094322	1	128	up	Ighv9-4	-
ENSMUSG00000094124	1	114	up	Ighv1-74	-
ENSMUSG00000094546	1	68	up	Ighv1-26	-

**Table A5:** Hubs identified in the network reconstructed from spleen *Ly6E*${}^{\Delta HSC}$ samples. Degree cutoff: 21. Reg. regulation

Ensembl ID	Cluster	Degree	Reg.	Symbol	Description
ENSMUSG00000027715	2	353	up	Ccna2	cyclin A2
ENSMUSG00000024742	3	349	up	Fen1	flap structure specific endonuclease 1
ENSMUSG00000024640	2	347	up	Psat1	phosphoserine aminotransferase 1
ENSMUSG00000040026	2	338	up	Saa3	serum amyloid A 3
ENSMUSG00000039713	2	327	down	Plekhg5	pleckstrin homology domain containing, family G (with RhoGef domain) member 5
ENSMUSG00000075289	4	322	down	Carns1	carnosine synthase 1
ENSMUSG00000067610	2	309	down	Klri1	killer cell lectin-like receptor family I member 1
ENSMUSG00000031503	1	305	up	Col4a2	collagen, type IV, alpha 2
ENSMUSG00000095700	3	298	up	Ighv10-3	-
ENSMUSG00000076613	3	287	up	Ighg2b	-
ENSMUSG00000051079	2	282	down	Rgs13	regulator of G-protein signaling 13
ENSMUSG00000036027	2	268	down	1810046K07Rik	RIKEN cDNA 1810046K07 gene
ENSMUSG00000027962	1	225	up	Vcam1	vascular cell adhesion molecule 1
ENSMUSG00000049130	1	184	up	C5ar1	complement component 5a receptor 1
ENSMUSG00000066861	1	35	up	Oas1g	2′-5′ oligoadenylate synthetase 1G

**Table A6:** Highly DEG. List of DEG that are differentially-expressed for every of the comparisons performed: control vs. cases at 3 d p.i., control vs. cases at 5 d p.i. and cases at 3 vs. 5 d p.i. Memb, membership to the group of samples genes belong; *ko*, *Ly6E*${}^{\Delta HSC}$ samples. Reg. Type refers to the three expression patterns observed, described in Section 5.

Ensembl ID	Symbol	Description	Memb.	Reg. Type
ENSMUSG00000032487	Ptgs2	prostaglandin-endoperoxide synthase 2	liver *wt*	1
ENSMUSG00000029816	Gpnmb	glycoprotein (transmembrane) nmb	liver *wt*	1
ENSMUSG00000035385	Ccl2	chemokine (C-C motif) ligand 2	liver *wt*	1
ENSMUSG00000035373	Ccl7	chemokine (C-C motif) ligand 7	liver *wt*	1
ENSMUSG00000015437	Gzmb	granzyme B	liver *wt*	1
ENSMUSG00000038037	Socs1	suppressor of cytokine signaling 1	liver *wt*	1
ENSMUSG00000026839	Upp2	uridine phosphorylase 2	liver *ko*	2
ENSMUSG00000075014	Gm10800	-	liver *ko*	1
ENSMUSG00000040660	Cyp2b9	cytochrome P450, family 2, subfamily b, polypeptide 9	liver *ko*	2
ENSMUSG00000056978	Hamp2	hepcidin antimicrobial peptide 2	liver *ko*	2
ENSMUSG00000073940	Hbb-bt	hemoglobin, beta adult t chain	liver *ko*	2
ENSMUSG00000052305	Hbb-bs	hemoglobin, beta adult major chain	liver *ko*	2
ENSMUSG00000025473	Adam8	a disintegrin and metallopeptidase domain 8	liver *ko*	1
ENSMUSG00000056973	Ces1d	carboxylesterase 1D	liver *ko*	2
ENSMUSG00000025317	Car5a	carbonic anhydrase 5a, mitochondrial	liver *ko*	2
ENSMUSG00000050578	Mmp13	matrix metallopeptidase 13	liver *ko*	1
ENSMUSG00000049723	Mmp12	matrix metallopeptidase 12	liver *ko*	1
ENSMUSG00000035878	Hykk	hydroxylysine kinase 1	liver *ko*	2
ENSMUSG00000069917	Hba-a2	hemoglobin alpha, adult chain 2	liver *ko*	2
ENSMUSG00000009350	Mpo	myeloperoxidase	liver *ko*	1
ENSMUSG00000109482	Gm4756	-	liver *ko*	2
ENSMUSG00000060807	Serpina6	serine (or cysteine) peptidase inhibitor, clade A, member 6	liver *ko*	2
ENSMUSG00000079018	Ly6c1	lymphocyte antigen 6 complex, locus C1	liver *ko*	1
ENSMUSG00000074896	Ifit3	interferon-induced protein with tetratricopeptide repeats 3	liver *ko*	3
ENSMUSG00000062488	Ifit3b	interferon-induced protein with tetratricopeptide repeats 3B	liver *ko*	3
ENSMUSG00000032808	Cyp2c38	cytochrome P450, family 2, subfamily c, polypeptide 38	liver *ko*	2
ENSMUSG00000025004	Cyp2c40	cytochrome P450, family 2, subfamily c, polypeptide 40	liver *ko*	2
ENSMUSG00000042248	Cyp2c37	cytochrome P450, family 2, subfamily c, polypeptide 37	liver *ko*	2
ENSMUSG00000067225	Cyp2c54	cytochrome P450, family 2, subfamily c, polypeptide 54	liver *ko*	2
ENSMUSG00000054827	Cyp2c50	cytochrome P450, family 2, subfamily c, polypeptide 50	liver *ko*	2
ENSMUSG00000001131	Timp1	tissue inhibitor of metalloproteinase 1	liver *ko*	1
ENSMUSG00000015437	Gzmb	granzyme B	spleen *wt*	1
ENSMUSG00000022584	Ly6c2	lymphocyte antigen 6 complex, locus C2	spleen *wt*	1
ENSMUSG00000040026	Saa3	serum amyloid A 3	spleen *ko*	1

## Appendix B. Validation of the Reconstruction Method

The reconstruction method employed in this case study was validated against other thee well-known inference methods: *ARACNe* [93], *WGCNA* [94] and *wTO* [95]. The output of each reconstruction method, using default values (including *EnGNet*) was compared to a gold standard (GS), retrieved from the *STRING* database.

Four different GSs were taken into consideration, since these were reconstructed from the DEG that were identified in the comparison of control vs. case samples at 5 d p.i., as shown in Section 4.2. These DEG were mapped to the *STRING* database gene identifiers selecting *Mus musculus* as model organism (taxid: 10090). A variable percentage of DEG (6–20%) could not be assigned to a STRING identifier, and were thus removed from the analysis. The interactions exclusively concerning the resulting DEG in each case were retrieved from the STRING database. These interaction networks would serve as GSs. The mentioned DEG (without unmapped identifiers) would also serve as input for the four reconstruction methods to be compared.

The *ARACNe* networks were inferred using the Spearman correlation coefficient following the implementations in the *minet* [96] *R* package. In this case, mutual information values were normalized and scaled in the range 0–1. On the other hand, the *WGCNA* networks were reconstructed following the original tutorial provided by the authors [97]. The power was defined as 5. Additionally, the *wTO* networks were built using Pearson correlation in accordance to the documentation. Absolute values were taken as relationship weights. Finally, *EnGNet* networks were inferred using the default parameters described in the original article by Gómez-Vela et al. [33]. For the comparison, the Receiver operating characteristic (ROC)-curve was estimated using the *pROC* [98] *R* package. ROC curves are shown in Figure A10.

The area under the ROC curve (AUC) was also computed in each case for the quantitative comparison of the methods, as shown in Figure A11a. The AUC compares the reconstruction quality of each method against random prediction. An AUC ≈ 1 corresponds to the perfect classifier whereas am AUC ≈ 0.5 approximates to a random classifier. Thus, the higher the AUC, the better the predictions. On average, *EnGNet* provided the best AUC results, whilst maintaining a good discovery rate. In addition, *EnGNet* provided relatively scarce networks compared to *WGCNA*, as shown in Figure A11b. This is considered of relevance given that sparseness is a main feature of gene networks [7].
